# De Ritis Ratio as an Independent Predictor of In-Hospital Mortality in Surgically Treated Acute Aortic Dissection

**DOI:** 10.3390/jcm15093419

**Published:** 2026-04-29

**Authors:** Kemal Eşref Erdoğan, Burak Kardeşler, Muhammet Fethi Sağlam, Emrah Uğuz, Murat Yücel, Servet Turgut, Mehmet Murat Yiğitbaşı, Mehmet Erdoğan, Kamuran Kalkan, Erol Şener

**Affiliations:** 1 Department of Cardiovascular Surgery, Ankara Yildirim Beyazit University, School of Medicine and Ankara Bilkent City Hospital, 06760 Ankara, Türkiye; dr.m.fethisaglam@gmail.com (M.F.S.); emrahuguz@gmail.com (E.U.); erolsene@gmail.com (E.Ş.); 2 Department of Cardiology, Ankara Bilkent City Hospital, 06800 Ankara, Türkiye; burak_kardesler@hotmail.com; 3 Department of Cardiovascular Surgery, Ankara Bilkent City Hospital, 06800 Ankara, Türkiye; dr_yucelmurat@hotmail.com; 4 Department of Cardiovascular Surgery, Kars Harakani State Hospital, 36100 Kars, Türkiye; drservetturgut@gmail.com; 5 Department of Cardiology, Ankara Yildirim Beyazit University, School of Medicine and Ankara Bilkent City Hospital, 06760 Ankara, Türkiye; mmuratyigitbasi@gmail.com (M.M.Y.); mhmterdogan@windowslive.com (M.E.); kamurankalkandr@gmail.com (K.K.)

**Keywords:** acute aortic dissection, De Ritis ratio, AST/ALT ratio, in-hospital mortality, risk stratification, cardiac surgery, biomarkers

## Abstract

**Background/Objectives:** Every hour of delay in treating acute aortic dissection (AAD) increases mortality by 1–2%, underscoring the critical need for rapid identification of high-risk patients. Despite advances in surgical management, in-hospital mortality remains substantial, and early, reliable risk stratification tools are urgently needed. We targeted to assess the relationship between the De Ritis ratio (DRr) and in-hospital mortality in patients experiencing surgery for AAD. **Methods:** In this single-center retrospective study, 182 patients who underwent surgery for AAD between 2020 and 2025 were included. Demographic, clinical, laboratory, and echocardiographic data were analyzed. Receiver operating characteristic analysis was used to estimate the DRr’s discriminatory ability, and univariable and multivariable logistic regression analyses were utilized to find predictors of in-hospital mortality. **Results:** In-hospital mortality occurred in 59 patients (32.4%). When comparing survivors to non-survivors, the DRr was noticeably greater. Along with age, lactate level, and ejection fraction, the DRr continued to be an independent predictor of in-hospital mortality in multivariable analysis. Receiver operating characteristic analysis showed moderate discriminatory performance of the DRr, with an area under the curve of 0.701 and an optimal cut-off value of 1.308. **Conclusions:** The DRr is a practical and accessible biomarker related to in-hospital mortality in surgically treated AAD and may provide incremental prognostic value when used alongside established clinical and laboratory parameters.

## 1. Introduction

Acute aortic dissection (AAD) is a fatal cardiovascular emergency that requires prompt diagnosis and treatment [1,2]. Despite advances in surgical and medical therapies, mortality remains elevated, particularly during the in-hospital period [3]. Consequently, early risk stratification remains essential. Prognostic factors in AAD include advanced age, hemodynamic instability, Stanford type A dissection, reduced ejection fraction, elevated lactate levels, and biochemical markers indicative of organ perfusion impairment [4,5,6]. Therefore, rapid, easily accessible laboratory parameters for early risk prediction have significant clinical value. While the De Ritis ratio (DRr) has emerged as a promising prognostic marker in other cardiovascular conditions, whether it provides independent and incremental prognostic value for predicting in-hospital mortality specifically in patients with acute type A AAD remains uncertain.

Aspartate aminotransferase (AST) and alanine aminotransferase (ALT) are liver enzymes generally assessed in daily biochemical evaluations. AST is present not only in hepatocytes but additionally in myocardium and skeletal muscle, whereas ALT is more liver specific [7]. The DRr (AST/ALT), stated as the ratio of these enzymes, serves as a biomarker reflecting systemic hypoperfusion, tissue hypoxia, and multiple organ dysfunction rather than isolated hepatocellular injury [8,9].

Contemporary studies have demonstrated that the DRr correlates with mortality and adverse clinical outcomes in several cardiovascular diseases, such as acute coronary syndrome (ACS), pulmonary embolism, and heart failure [10]. For context, the discriminatory power of the DRr in predicting mortality has shown area under the curve (AUC) fluctuating from 0.68 to 0.75 across acute vascular syndromes such as myocardial infarction and pulmonary embolism in prior research. Based on this comparative evidence, we hypothesized that the DRr would achieve an AUC of at least 0.70 for predicting in-hospital mortality in AAD, allowing us to judge its prognostic impact within a clinically meaningful range.

However, in the setting of AAD—a complex condition with high mortality—the prognostic value of the DRr has been researched in a limited number of studies, and available data remain insufficient [11].

This study evaluates the association among the DRr and in-hospital mortality among patients undergoing surgery for type 1 AAD and assesses the prognostic value of this ratio.

## 2. Materials and Method

**Figure 1:**
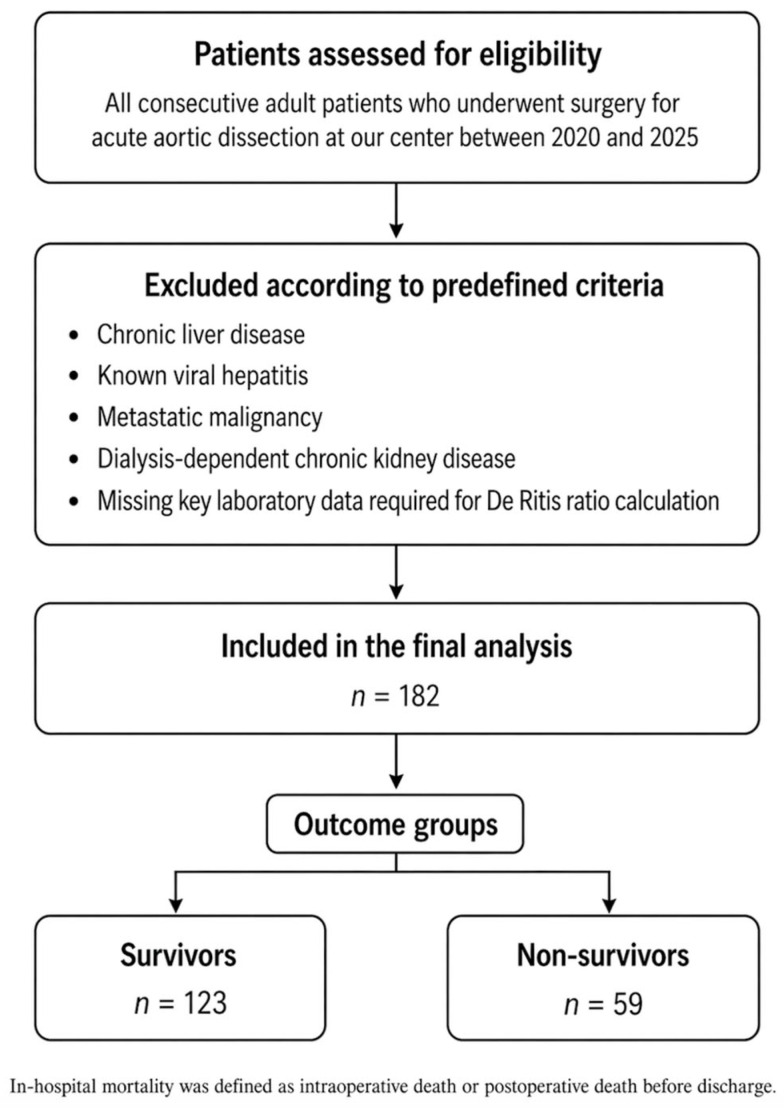
Study flow diagram.

Our retrospective and single center study analyzed data from 182 consecutive adult patients who underwent surgery for AAD between 2020 and 2025. Given the retrospective design of the study, no formal a priori sample size calculation was performed A comprehensive review of hospital medical records was performed to obtain data on demographic characteristics, clinical status, laboratory measurements, and echocardiographic parameters. Adults aged 18 years and above who underwent surgical treatment for AAD were eligible for inclusion. Key exclusion criteria were chronic liver disease, known viral hepatitis, metastatic malignancy, and chronic kidney disease requiring dialysis, as these conditions could confound the assessment of the DRr. Patients with missing key laboratory data required to calculate the DRr were also excluded. This approach aimed to ensure the reliability and reproducibility of the findings by minimizing potential confounding influences on aminotransferase levels. Figure 1 presents the study flow diagram.

### 2.1. Definitions

In-hospital mortality referred to any death that occurred during the operation or at any time after surgery before discharge. The DRr was determined by dividing the AST level by the ALT level based on laboratory values obtained at admission.

### 2.2. Clinical and Laboratory Assessment

Routine laboratory parameters at admission, including complete blood count, biochemical tests, and inflammatory markers, were evaluated for all patients. Complete blood count parameters were measured using an automated hematology analyzer (XN-1000, Sysmex Corporation, Kobe, Japan), and biochemical parameters, including AST and ALT, were measured using an automated biochemical analyzer (Cobas 8000, Roche Diagnostics, Mannheim, Germany). Echocardiographic examinations recorded ejection fraction and aortic valve pathologies. The dissection type was classified according to the Stanford system. Operative details and perioperative clinical information were gathered from the patients’ documented hospital records.

Operative variables, including time from symptom onset to surgery, cross-clamp duration, cardiopulmonary bypass duration, and antegrade cerebral perfusion time, were obtained from surgical records and evaluated in relation to in-hospital mortality.

### 2.3. Perioperative Management

Preoperative management in patients with acute AAD was focused on rapid stabilization and prevention of rupture. All patients were monitored closely, with prompt blood pressure and heart rate control, primarily using esmolol; nitrates were added when needed. Analgesia was provided to reduce sympathetic activation, most commonly with morphine. Emergency blood product preparation and close coordination among the emergency department, intensive care unit, anesthesia team, and operating room were ensured.

General anesthesia was induced in a controlled manner to avoid abrupt hypertension and tachycardia, which could aggravate dissection propagation or precipitate rupture. Intraoperative monitoring included invasive arterial pressure monitoring, central venous catheterization, urinary catheterization, and near-infrared spectroscopy (NIRS) for continuous cerebral oxygenation monitoring in all patients. Near-infrared spectroscopy was performed using a NIRS monitoring system (INVOS 5100C, Medtronic, Minneapolis, MN, USA). A decrease of more than 20% from baseline cerebral oxygen saturation was considered clinically significant and prompted corrective measures. Anticoagulation was administered with careful dose adjustment and activated clotting time monitoring.

Our routine cerebral protection strategy generally involved high–moderate hypothermia, typically in the 24–28 °C range, together with antegrade cerebral perfusion during the distal aortic anastomosis. In a limited number of patients, total circulatory arrest under deep hypothermia was required; in these cases, cooling to approximately 18 °C was used. Additional pharmacologic neuroprotection during antegrade cerebral perfusion included propofol or thiopental together with methylprednisolone, along with mild hypercapnia to facilitate cerebral vasodilation, according to institutional practice. When indicated by intraoperative monitoring, corrective measures included adjustment of cannulation and flow, optimization of head and neck position, maintenance of hemoglobin level, and conversion to bilateral antegrade cerebral perfusion.

Perioperative transfusion of blood products was performed according to bleeding severity, hemodynamic status, and laboratory parameters. Variables related to perioperative management, including cardioplegia type, cooling temperature, blood product use, drainage volume, duration of mechanical ventilation, and intensive care and hospital length of stay, were recorded.

### 2.4. Statistical Analysis

All statistical evaluations were performed with IBM SPSS Statistics for Windows, version 25.0 (IBM Corp., Armonk, NY, USA). The spreading of continuous variables was measured visually and with normality tests (Shapiro–Wilk). Non-normally distributed continuous variables were stated as median (interquartile range), while categorical variables were given as counts and percentages.

For the comparison of survivors and non-survivors, continuous variables were explored with the Mann–Whitney U test, whereas categorical variables were assessed using the chi-square or Fisher’s exact test. Univariable logistic regression identified variables related to in-hospital mortality. Factors showing a significant suggestion in the univariable analysis (*p* < 0.05), together with variables considered clinically important, were entered into the multivariable logistic regression model. The findings are reported as odds ratios with corresponding 95% confidence intervals.

ROC analysis was executed to decide how well the DRr distinguished patients who experienced in-hospital death from those who did not. The AUC was testified with corresponding 95% confidence intervals. The most appropriate threshold value was identified using the Youden index, which was selected for its standard ability to balance sensitivity and specificity, thereby optimizing overall diagnostic accuracy for urgent clinical triage. This approach was preferred over relying solely on predefined clinical thresholds to ensure that both false negatives and false positives were minimized in the context of high-stakes decision-making. A *p* value below 0.05 was studied suggestive of statistical significance in all analyses.

## 3. Results

Overall, 182 patients treated surgically for AAD were involved in the analysis. In-hospital mortality happened in 59 patients (32.4%). Compared to survivors, non-survivors were older and exhibited significantly higher creatinine and lactate levels, as well as lower ejection fraction. Most demographic characteristics and comorbidities did not differ significantly between groups. Baseline clinical, laboratory, and echocardiographic characteristics are detailed in Table 1.

The median DRr was 1.17 (0.94–1.47) in survivors and 1.47 (1.23–2.42) in non-survivors. The observed difference reached statistical significance (*p* < 0.001). Detailed intergroup comparisons of laboratory measurements, including the DRr, are revealed in Table 1.

Analysis using univariable logistic regression revealed a significant association between the DRr and in-hospital mortality. Each 1-unit increase in the DRr corresponded to an approximately 2.4-fold rise in mortality risk (OR: 2.41; 95% CI: 1.54–3.76; *p* < 0.001). To provide a clearer sense of the clinical impact, for example, patients with a DRr of 1.0 had an estimated in-hospital mortality risk of 20%, while those with a ratio of 2.0 had an estimated risk of 39%. This illustrates how higher DRrs are allied with a marked increase in absolute mortality risk.

In addition, increasing age, elevated serum lactate levels, and decreased ejection fraction were all found to be significantly related to in-hospital mortality. The findings of the univariable logistic regression analysis are summarized in Table 2.

Analysis using univariable logistic regression revealed a significant association between the De Ritis ratio (DRr) and in-hospital mortality. Each 1-unit increase in the DRr was associated with an approximately 2.4-fold increase in the risk of in-hospital mortality (OR: 2.41; 95% CI: 1.54–3.76; *p* < 0.001). In addition, increasing age, elevated serum lactate levels, and lower left ventricular ejection fraction were significantly associated with in-hospital mortality. The results of the univariable logistic regression analysis are summarized in Table 2.

To assess the independent prognostic value of the DRr, a multivariable logistic regression model was constructed including variables that were significant in the univariable analysis together with clinically relevant covariates. Variable selection was based on both statistical significance and clinical relevance while avoiding overfitting. All selected variables were entered simultaneously using the enter method. Multicollinearity was assessed by variance inflation factor analysis, and no relevant collinearity was detected among the predictors (all VIFs < 2). The multivariable model was statistically significant overall and showed acceptable calibration. Within this model, the DRr remained an independent predictor of in-hospital mortality (OR: 2.43; 95% CI: 1.44–4.12; *p* = 0.001), together with age, lactate level, and left ventricular ejection fraction, whereas coronary artery disease did not retain statistical significance after adjustment. The detailed results of the multivariable analysis are presented in Table 2.

Operative and perioperative characteristics according to in-hospital mortality are summarized in Table 3. Compared with survivors, non-survivors had a significantly shorter time from symptom onset to surgery; longer cross-clamp, cardiopulmonary bypass, and antegrade cerebral perfusion times; greater erythrocyte suspension and fresh frozen plasma requirements; higher drainage volume; and longer extubation times. Cooling temperature, platelet transfusion, and cryoprecipitate use did not differ significantly between groups. ICU and total hospital stay were shorter in non-survivors, likely reflecting earlier death during the postoperative course. Total circulatory arrest under deep hypothermia was performed in four patients, with cooling to approximately 18 °C; three of these patients survived, whereas one died during the postoperative period.

**Table 3:** Operative and perioperative characteristics according to in-hospital mortality.

Variable	Survivors (n = 123)	Non-Survivors (n = 59)	*p*-Value
Time from symptom onset to surgery, h	11 (8–18)	8 (4.5–12.5)	0.002
Cross-clamp time, min	111 (93–134)	140 (119–156)	<0.001
Cardiopulmonary bypass time, min	158 (134–183.5)	190 (172.5–217.5)	<0.001
Antegrade cerebral perfusion time, min	31 (24–39)	39 (32–46)	<0.001
Cooling temperature, °C	28 (26–28)	28 (26–28)	0.894
Erythrocyte suspension use, units	3 (1–4)	4 (2–5)	0.001
Fresh frozen plasma, units	2 (0–3)	2 (1.5–4)	0.014
Platelet transfusion, units	0 (0–0)	0 (0–0.5)	0.131
Cryoprecipitate, units	0 (0–0)	0 (0–0)	0.351
Total drainage, mL	950 (675–1375)	1150 (825–1875)	0.010
Extubation time, h	12 (9–18)	26 (17–48)	<0.001
ICU stay, days	3 (2–4)	2 (1–3)	<0.001
Hospital stay, days	8 (7–11)	3 (2–4.5)	<0.001

Abbreviations: ICU: Intensive care unit. In an extended multivariable model including rupture and pericardial hematoma, the DRr retained its prognostic significance (OR: 2.43; 95% CI: 1.44–4.12; *p* = 0.001). A complete summary of the outcomes is presented in Table 2. The discriminatory power of the DRr in predicting in-hospital mortality was calculated by ROC analysis. The area under the curve (AUC) for the DRr was calculated as 0.701 (95% CI: 0.618–0.787). The optimal cut-off value, determined by the Youden index, was 1.308. Using this threshold value, sensitivity was 69.5%, and specificity was 62.6%. An AUC of 0.701 reflects moderate prognostic utility in clinical practice; while values above 0.80 are generally considered suitable for “rule-in” or “rule-out” purposes, moderate AUCs in the range of 0.70 to 0.80, as seen here, indicate meaningful but not definitive risk stratification. This aligns with moderate prognostic utility akin to established biomarkers such as lactate. The ROC curve and cut-off analysis results are shown in Figure 2.

**Figure 2:**
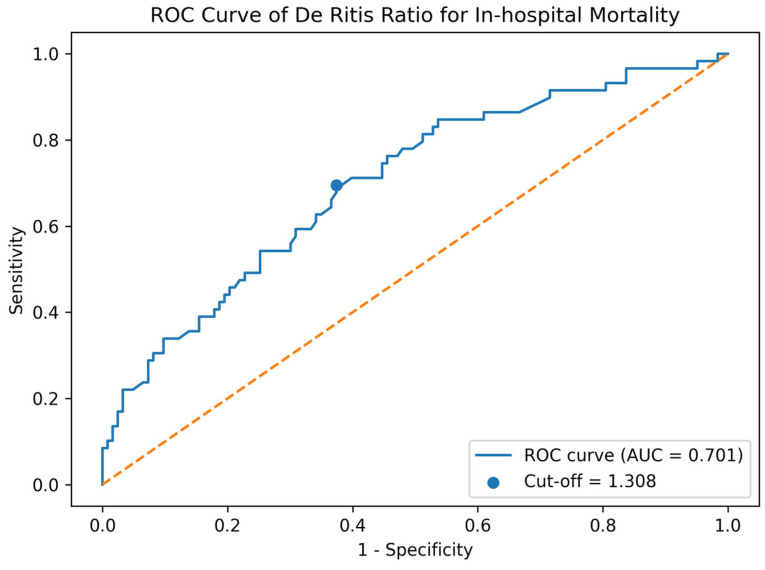
ROC analysis of the De Ritis ratio in predicting in-hospital mortality. (ROC: Receiver Operating Characteristic).

## 4. Discussion

The ends of this study suggest that an elevated DRr (AST/ALT) is independently related to in-hospital mortality among individuals undergoing operative management for AAD. The significantly higher DRr observed in non-survivors compared to survivors suggests that this biochemical parameter may indicate poor prognosis in AAD. Notably, during AAD, episodes of dynamic malperfusion or perioperative low-flow states can result in myocardial ischemia and hepatocellular hypoxia, both of which promote the release of AST into the circulation. This mechanism strengthens the biological plausibility of the observed association. Furthermore, the finding that each 1-unit increase in the DRr is related to approximately a 2.4-fold rise in mortality risk in univariable logistic regression underscores the clinical relevance of this association.

The inclusion of operative variables provides additional clinical context for the interpretation of our findings. In the present cohort, non-survivors had a shorter time from symptom onset to surgery and longer cross-clamp, cardiopulmonary bypass, and antegrade cerebral perfusion times compared with survivors. These findings are consistent with the greater procedural complexity and hemodynamic burden observed in higher-risk patients. At the same time, they support the view that the prognostic value of the De Ritis ratio should be interpreted alongside operative factors rather than in isolation.

The potential prognostic role of the DRr has been increasingly recognized in numerous cardiovascular diseases. Ndrepepa et al. informed that a higher DRr was an independent predictor of short-term mortality among patients with ACS [12]. Similarly, Ewid et al. reported its effectiveness in envisaging both in-hospital and long-term mortality in heart failure patients [13]. Additional studies have considered the DRr in diverse clinical contexts. For instance, Steininger et al. identified it as an independent prognostic factor for long-term mortality following acute myocardial infarction [14]. In a retrospective analysis of patients with COVID-19, an elevated DRr at admission was significantly associated with increased in-hospital mortality and demonstrated measurable discriminatory ability in ROC analysis [15]. Furthermore, a large trauma patient study reported markedly increased mortality risk associated with elevated DRrs [16]. These findings imply that, beyond the predictive value of AST or ALT alone, the DRr may reveal systemic stress, hypoperfusion, and organ dysfunction, thereby associating with mortality in various acute and critical conditions.

In the context of AAD, the literature is more limited. Existing studies have mainly focused on lactate, D-dimer, and inflammatory markers, and the prognostic value of liver enzyme ratios has not been sufficiently investigated [17,18]. In this respect, our study contributes to the literature as one of the few demonstrating an association between the DRr and in-hospital mortality in AAD.

The prognostic significance of an elevated DRr is multifactorial. AST is present in hepatocytes, myocardium, and skeletal muscle, whereas ALT more specifically indicates hepatocellular injury. Hemodynamic instability, reduced cardiac output, and systemic hypoxia during AAD may cause a disproportionate increase in AST levels. Therefore, an elevated DRr likely reflects multiple organ involvement and circulatory disturbances rather than isolated liver injury [9]. The sustained significance of the DRr alongside established prognostic markers such as lactate and ejection fraction in multivariable analyses supports its role as an independent indicator of AAD severity.

The moderate discriminatory performance of the DRr in ROC analysis (AUC: 0.701) should be interpreted in the context of AAD, a condition characterized by complex and multifactorial pathophysiology in which even established risk models demonstrate comparable performance. Rather than serving as a standalone risk stratification tool, the DRr may provide meaningful incremental value when integrated with existing clinical and laboratory parameters.

Although a cut-off value of 1.308 was identified in our analysis, its clinical applicability may be influenced by population-specific factors. Therefore, the DRr may be more appropriately interpreted as a continuous variable, while still offering potential utility for early identification of higher-risk patients and guiding closer clinical monitoring.

Although studies evaluating the prognostic value of the DRr in patients with AAD are limited, some indirect evidence exists. A risk model derived from a large multicenter Chinese cohort identified the DRr as one of the six most important prognostic variables, alongside eGFR, leukocyte count, platelet count, hemoglobin, and albumin [9,19,20,21]. However, the primary endpoint of that study was multiple organ dysfunction syndrome rather than mortality. In contrast, the present study specifically focuses on in-hospital mortality and preoperative risk stratification in patients with acute Stanford type A aortic dissection. Moreover, prior studies have generally evaluated aminotransferases separately, without considering the DRr (AST/ALT) as a composite marker preoperative risk assessment, and the association of liver enzymes with early mortality [22]. This distinction is clinically relevant, as the DRr may better reflect the combined effects of systemic hypoperfusion and multiorgan involvement compared to isolated liver enzyme measurements.

Kimura et al. recently proposed a prognostic score to predict early in-hospital mortality in patients with acute thoracic aortic dissection. This scoring system incorporated AST-derived biomarkers but did not include the DRr directly. Nonetheless, the association of AST-based parameters with early mortality supports the prognostic value of aminotransferase-derived indicators in AAD [23].

This study differs from previous research in several key aspects. First, the DRr was evaluated as the primary predictor rather than as a secondary variable or score component. Second, the endpoint was a clear and clinically meaningful outcome: in-hospital mortality. Finally, the prognostic value of the DRr was assessed using a classical, transparent logistic regression approach, thereby enhancing the clinical interpretability of the results.

In addition to the independent prognostic value of the De Ritis ratio, other variables identified in the multivariable analysis also deserve consideration. Age, lactate level, and left ventricular ejection fraction were independently associated with in-hospital mortality. These findings are clinically and pathophysiologically plausible. Advanced age is a well-established determinant of adverse outcome in AAD, likely reflecting reduced physiological reserve, a greater burden of comorbidities, and increased susceptibility to perioperative complications [24,25]. Elevated lactate levels are widely recognized as a marker of systemic hypoperfusion and circulatory failure, whereas lower left ventricular ejection fraction may indicate impaired cardiac reserve and reduced tolerance to the profound hemodynamic stress associated with AAD surgery.

The relationship between the DRr and lactate is particularly noteworthy, as both markers may provide insight into hypoperfusion, although through partially different mechanisms. Lactate primarily reflects impaired tissue oxygen delivery and anaerobic metabolism, whereas the DRr may represent the biochemical consequences of hypoperfusion-related organ injury, including hepatocellular and myocardial stress [26]. Therefore, these two markers may capture overlapping but non-identical dimensions of circulatory compromise. The persistence of both variables in the prognostic model suggests that the DRr may provide complementary prognostic information beyond lactate alone.

From a clinical perspective, the DRr may represent a practical, inexpensive, and readily available biomarker for early risk assessment in patients undergoing surgery for AAD. Because AST and ALT are routinely measured in standard biochemical testing, the DRr can be calculated without additional cost or delay. Although it should not be considered a standalone predictor, its independent association with in-hospital mortality suggests that it may support clinicians in identifying higher-risk patients, refining perioperative risk stratification, and guiding closer monitoring and more individualized management strategies.

An important advantage of this study is its relatively large cohort of 182 patients, especially in the context of a rare and highly fatal condition such as AAD. In addition, the occurrence of mortality in 59 patients enabled reliable multivariable regression analyses. Demonstrating the prognostic value of an easily accessible, low-cost parameter, such as the DRr, enhances the clinical applicability of the study. It should be acknowledged that the data were derived from a single-center cohort, which may limit the generalizability of the conclusions to broader patient populations or different healthcare settings.

Several limitations of this study should be acknowledged. First, its retrospective design and single-center setting may have increased the risk of selection bias and may limit the generalizability of the findings. Second, the DRr was calculated solely from admission laboratory values and therefore did not account for dynamic changes over time; serial measurements, including perioperative and postoperative assessments, may provide additional prognostic information by reflecting the temporal progression of systemic hypoperfusion and organ dysfunction. Although several operative and perioperative variables were available and are now presented, they were not fully incorporated into the primary prognostic analysis. Parameters such as aortic cross-clamp duration, cooling temperature, transfusion-related variables, and drainage volume may provide additional prognostic information. However, some relevant data, including the exact duration of hypothermia and standardized perioperative risk scores, were not systematically available. Therefore, the effects of different temperature management strategies and the incremental prognostic value of certain intraoperative variables could not be comprehensively evaluated. In addition, postoperative ICU-based risk scores such as the Sequential Organ Failure Assessment (SOFA) score were not available in our dataset, precluding direct comparison between the admission DRr and established postoperative scoring systems. Likewise, a formal comparison between the DRr and established preoperative surgical risk scores such as EuroSCORE II was not performed. Future studies integrating serial DRr measurements with operative characteristics, perioperative variables, and validated preoperative and postoperative risk scores may provide a more comprehensive and clinically applicable model for risk stratification in AAD. Nevertheless, adjustment for potential confounders through multivariable analysis strengthens the validity of the findings.

## 5. Conclusions

Overall, the DRr represents a practical and clinically informative biomarker for the prediction of in-hospital mortality among patients with AAD. However, its moderate discriminatory power indicates that it should not be used as a standalone risk determinant but rather as a complementary biomarker alongside existing clinical, hemodynamic, and laboratory indicators. This integrated approach may enhance patient management, particularly in multidisciplinary decision-making.

## Figures and Tables

**Table 1:** Baseline characteristics according to in-hospital mortality.

Variable	Survivors	Non-Survivors	*p*-Value
Age	57 (45–65)	62 (55.5–70)	0.001
Albumin	41 (38–43)	41 (38–42)	0.279
Creatinine	0.97 (0.80–1.18)	1.04 (0.91–1.25)	0.042
D-dimer	9.66 (2.01–29.13)	8.91 (2.35–31.31)	0.964
WBC	11.40 (9.23–13.89)	12.07 (8.99–15.29)	0.496
Hemoglobin	13.30 (12.15–14.65)	13.40 (12–14.65)	0.956
Platelet	209 (172–253)	198 (157–260)	0.291
AST	26 (20–40)	42 (25–82.50)	03
ALT	23 (17–32)	22 (17–40.50)	0.555
CRP	6.80 (1–19.55)	5 (1–17.73)	0.843
Ph	7.40 (7.36–7.45)	7.37 (7.29–7.45)	0.042
Lactate	2.39 (1.31–3.52)	2.78 (1.54–5.52)	0.081
LVEF, %	55 (50–60)	55 (50–55)	0.050
Aortic Diameter	50 (46–56)	54 (47–55)	0.437
De Ritis	1.17 (0.94–1.47)	1.47 (1.23–2.42)	<0.001
Gender (male), n (%)	91 (74.0)	37 (62.7)	0.166
HT, n (%)	96 (78.0)	41 (69.5)	0.285
DM, n (%)	36 (29.3)	21 (35.6)	0.489
COPD, n (%)	9 (7.3)	2 (3.4)	0.507
CAD, n (%)	18 (14.6)	22 (37.3)	0.001
Type 1, n (%)	91 (74.0)	44 (74.6)	N/A
Type 2, n (%)	32 (26.0)	16 (27.1)	0.929
Intramural Hematoma, n (%)	11 (8.9)	7 (11.9)	0.724
Pericardial hematoma, n (%)	19 (15.4)	10 (16.9)	0.965
Rupture, n (%)	12 (9.8)	11 (18.6)	0.146

Abbreviations: WBC: White blood count, AST: Aspartate Aminotransferase, ALT: Alanine Aminotransferase, CRP: C-Reactive Protein, LVEF: Left ventricular ejection fraction, HT: Hypertension, DM: Diabetes mellitus, COPD: Chronic obstructive pulmonary disease, CAD: Coronary artery disease.

**Table 2:** Univariable and multivariate logistic regression analysis for in-hospital mortality.

Variable	Univariable OR (95% CI)	*p*-Value	Multivariable OR (95% CI)	*p*-Value
De Ritis ratio	2.41 (1.54–3.76)	<0.001	2.43 (1.44–4.12)	0.001
Gender	0.59 (0.30–1.15)	0.121	-	-
pH	0.01 (0.00–0.38)	0.054	-	-
Albumin	0.95 (0.89–1.02)	0.166	-	-
Hypertension	0.64 (0.32–1.29)	0.212	-	-
White blood cell count	1.04 (0.97–1.12)	0.294	-	-
Platelet count	1.00 (0.99–1.00)	0.298	-	-
COPD	0.44 (0.09–2.13)	0.309	-	-
Aortic diameter	1.02 (0.98–1.05)	0.334	-	-
Left ventricular ejection fraction	0.95 (0.91–1.00)	0.038	0.94 (0.88–0.99)	0.029
Diabetes mellitus	1.34 (0.69–2.58)	0.389	-	-
CRP	1.00 (0.99–1.00)	0.396	-	-
Lactate	1.20 (1.06–1.36)	0.045	1.21 (1.03–1.42)	0.019
Creatinine	1.10 (0.85–1.43)	0.469	-	-
Intramural hematoma	1.37 (0.50–3.74)	0.537	-	-
Age	1.05 (1.02–1.08)	0.008	1.05 (1.01–1.08)	0.011
Pericardial hematoma	1.12 (0.48–2.58)	0.795	-	-
Coronary artery disease	3.47 (1.68–7.18)	0.008	2.24 (0.88–5.70)	0.090
Hemoglobin	1.02 (0.88–1.18)	0.802	-	-
Type 1 dissection	1.03 (0.51–2.10)	0.931	-	-

Abbreviations: COPD: Chronic obstructive pulmonary disease, CRP: C-Reactive Protein.

## Data Availability

The data presented in this study are available on request from the corresponding author.

## Abbreviations

The following abbreviations are used in this manuscript:

AAD	Acute aortic dissection
DRr	De Ritis ratio
AST	Aspartate aminotransferase
ALT	Alanine aminotransferase
ACS	Acute coronary syndrome
ROC	Receiver Operating Characteristic
AUC	Area under the curve

## References

[1] Leivaditis V., Ozsoy E., Dahm M., Papatriantafyllou A., Buki T., Baikoussis N.G. (2025). From Cardiac Arrest to Survival: Managing Acute Type A Aortic Dissection With Emergent Surgery. Cureus.

[2] Belyaev A.M. (2025). A Comprehensive Review of Acute Type A Aortic Dissection: Epidemiology, Classification, Management Strategies, Mortality Risk Assessment, and Ethical Considerations for Patients who Refuse Blood Transfusions. Rev. Cardiovasc. Med..

[3] Tzikas S., Loufopoulos G., Evangeliou A.P., Boulmpou A., Fragakis N., Vassilikos V. (2021). Acute aortic dissection type A: Case series and insights on incidence, management and outcomes. Hippokratia.

[4] Chukwu M., Ehsan P., Aburumman R.N., Muthanna S.I., Menon S.R., Vithani V., Sutariya B., Montenegro D.M., Mohammed L. (2023). Acute Stanford Type A Aortic Dissection: A Review of Risk Factors and Outcomes. Cureus.

[5] Kovoor J.G., Glynatsis J.M., Glynatsis N.C., Perrotta D., Chan E., Daniell T., Bacchi S., Stretton B., Tyagi D., Hewitt J.N. (2025). Factors affecting acute aortic dissection mortality: A multicentre cohort study. Surg. Pract. Sci..

[6] Rogers A.M., Hermann L.K., Booher A.M., Nienaber C.A., Williams D.M., Kazerooni E.A., Froehlich J.B., O’Gara P.T., Montgomery D.G., Cooper J.V. (2011). Sensitivity of the aortic dissection detection risk score, a novel guideline-based tool for identification of acute aortic dissection at initial presentation: Results from the international registry of acute aortic dissection. Circulation.

[7] Han J.H., Kwak J.Y., Lee S.S., Kim H.G., Jeon H., Cha R.R. (2022). Markedly Elevated Aspartate Aminotransferase from Non-Hepatic Causes. J. Clin. Med..

[8] Rief P., Pichler M., Raggam R., Hafner F., Gerger A., Eller P., Brodmann M., Gary T. (2016). The AST/ALT (De-Ritis) ratio: A novel marker for critical limb ischemia in peripheral arterial occlusive disease patients. Medicine.

[9] Wang L., Xu Y., Zhang S., Bibi A., Xu Y., Li T. (2022). The AST/ALT Ratio (De Ritis Ratio) Represents an Unfa-vorable Prognosis in Patients in Early-Stage SFTS: An Observational Cohort Study. Front. Cell Infect. Microbiol..

[10] Durak K., Nubbemeyer K., Zayat R., Spillner J., Dineva S., Kalverkamp S., Kersten A. (2024). De Ritis Ratio to Predict Clinical Outcomes of Intermediate- and High-Risk Pulmonary Embolisms. J. Clin. Med..

[11] Liu H., Diao Y.F., Shao Y.F., Qian S.C., Zeng Z.H., Fan G.L., Ma L.Y., Zhang H.J., on the behalf of the Additive Anti-inflammatory Action for Aortopathy & Arteriopathy (5A) Investigators (2024). Prognostic implication of residual inflammatory trajectories in acute type I aortic dissection: Dual-center prospective cohort study. Int. J. Surg..

[12] Ndrepepa G., Holdenrieder S., Kastrati A. (2022). Prognostic value of De Ritis ratio in patients with acute myocardial infarction. Clin. Chim. Acta.

[13] Ewid M., Sherif H., Allihimy A.S., Alharbi S.A., Aldrewesh D.A., Alkuraydis S.A., Abazid R. (2020). AST/ALT ratio predicts the functional severity of chronic heart failure with reduced left ventricular ejection fraction. BMC Res. Notes.

[14] Steininger M., Winter M.P., Reiberger T., Koller L., El-Hamid F., Forster S., Schnaubelt S., Hengstenberg C., Distelmaier K., Goliasch G. (2018). De-Ritis Ratio Improves Long-Term Risk Prediction after Acute Myocardial Infarction. J. Clin. Med..

[15] Dracz B., Czompa D., Mullner K., Hagymasi K., Miheller P., Szekely H., Papp V., Horvath M., Hritz I., Szijarto A. (2022). The Elevated De Ritis Ratio on Admission Is Independently Associated with Mortality in COVID-19 Patients. Viruses.

[16] Lin Y.C., Tsai C.H., Su W.T., Hsu S.Y., Hsieh C.H., Lin C.H. (2025). De Ritis Ratio as a Prognostic Marker for Mortality in Moderate-to-Severe Traumatic Brain Injury: A Propensity Score-Matched Analysis. Diagnostics.

[17] Qiu L., Ji Q., Miao H., Long M., Gong M., Ye J., Liu T., Zhang T., Huang Z., Liu Y. (2025). Prognostic Value of D-Dimer in Acute Type A Aortic Dissection and Intramural Hematoma: Observations from the Acute Aortic Syn-drome Group of the Registry of Acute Non-Traumatic Chest Pain in China. J. Am. Heart Assoc..

[18] Nappi F., Alzamil A., Salsano A., Avtaar Singh S.S., Gambardella I., Santini F., Fiore A., Perocchio G., Demondion P., Mesnildrey P. (2023). Lactate-Based Difference as a Determinant of Outcomes following Surgery for Type A Acute Aortic Dissection: A Multi-Centre Study. J. Clin. Med..

[19] Alvarez A.M., Mukherjee D. (2011). Liver abnormalities in cardiac diseases and heart failure. Int. J. Angiol..

[20] Sungur M.A., Sungur A., Zencirci A.E. (2023). The Relationship between the Presence of Cardiohepatic Syndrome and Mortality in Heart Failure with Reduced Ejection Fraction. Turk Kardiyol. Dern. Ars..

[21] Karatas M., Keles N., Parsova K.E., Ciftci H.O., Ozkok S., Kahraman E., Durak F., Kocogullari C.U., Yiyit N. (2023). High AST/ALT Ratio Is Associated with Cardiac Involvement in Acute COVID-19 Patients. Medicina.

[22] Kuang J., Yang J., Wang Q., Yu C., Li Y., Fan R. (2020). A preoperative mortality risk assessment model for Stanford type A acute aortic dissection. BMC Cardiovasc. Disord..

[23] Kimura S., Sato H., Shimajiri S., Nakayama T. (2025). An acute aortic dissection prognostic score for predicting early in-hospital mortality in acute thoracic aortic dissection. Am. Heart J. Plus.

[24] Ohnuma T., Shinjo D., Fushimi K. (2016). Hospital mortality of patients aged 80 and older after surgical repair for type A acute aortic dissection in Japan. Medicine.

[25] Wang J.C., Jacquemyn X., Sultan I., Serna-Gallegos D., Ahmad D., Bonatti J., Kaczorowski D., Chu D., Ogami T., Hasan I. (2026). Impact of Age on Survival After Acute Type A Aortic Dissection Repair. Ann. Vasc. Surg..

[26] Puluca N., König C., Wiesner G., Waschulzik B., Vitanova K., Krane M., Böhm J. (2025). Preoperative Hyperlactatemia Predicts Mortality in Acute Stanford Type A Dissection: A 16-Year-Period, Single-Center, Retrospective Study. J. Clin. Med..

